# Circulating Plasma Levels of miR-20b, miR-29b and miR-155 as Predictors of Bevacizumab Efficacy in Patients with Metastatic Colorectal Cancer

**DOI:** 10.3390/ijms19010307

**Published:** 2018-01-20

**Authors:** Paola Ulivi, Matteo Canale, Alessandro Passardi, Giorgia Marisi, Martina Valgiusti, Giovanni Luca Frassineti, Daniele Calistri, Dino Amadori, Emanuela Scarpi

**Affiliations:** 1 Biosciences Laboratory, Istituto Scientifico Romagnolo per lo Studio e la Cura dei Tumori (IRST) IRCCS, 47014 Meldola, Italy; matteo.canale@irst.emr.it (M.C.); giorgia.marisi@irst.emr.it (G.M.); daniele.calistri@irst.emr.it (D.C.); 2 Department of Medical Oncology, Istituto Scientifico Romagnolo per lo Studio e la Cura dei Tumori (IRST) IRCCS, 47014 Meldola, Italy; alessandro.passardi@irst.emr.it (A.P.); martina.valgiusti@irst.emr.it (M.V.); luca.frassineti@irst.emr.it (G.L.F.); dino.amadori@irst.emr.it (D.A.); 3 Unit of Biostatistics and Clinical Trials, Istituto Scientifico Romagnolo per lo Studio e la Cura dei Tumori (IRST) IRCCS, 47014 Meldola, Italy; emanuela.scarpi@irst.emr.it

**Keywords:** plasma, miRNAs, colorectal cancer, bevacizumab

## Abstract

Targeting angiogenesis in the treatment of colorectal cancer (CRC) is a common strategy, for which potential predictive biomarkers have been studied. miRNAs are small non-coding RNAs involved in several processes including the angiogenic pathway. They are very stable in biological fluids, which turns them into potential circulating biomarkers. In this study, we considered a case series of patients with metastatic (m) CRC treated with a bevacizumab (B)-based treatment, enrolled in the prospective multicentric Italian Trial in Advanced Colorectal Cancer (ITACa). We then analyzed a panel of circulating miRNAs in relation to the patient outcome. In multivariate analysis, circulating basal levels of hsa-miR-20b-5p, hsa-miR-29b-3p and hsa-miR-155-5p resulted in being significantly associated with progression-free survival (PFS) (*p* = 0.027, *p* = 0.034 and *p* = 0.039, respectively) and overall survival (OS) (*p* = 0.044, *p* = 0.024 and *p* = 0.032, respectively). We also observed that an increase in hsa-miR-155-5p at the first clinical evaluation was significantly associated with shorter PFS (HR 3.03 (95% CI 1.06–9.09), *p* = 0.040) and OS (HR 3.45 (95% CI 1.18–10.00), *p* = 0.024), with PFS and OS of 9.5 (95% CI 6.8–18.7) and 15.9 (95% CI 8.4–not reached), respectively, in patients with an increase ≥30% of hsa-miR-155-5p and 22.3 (95% CI 10.2–25.5) and 42.9 (24.8–not reached) months, respectively, in patients without such increase. In conclusion, our results highlight the potential usefulness of circulating basal levels of hsa-miR-20b-5p, hsa-miR-29b-3p and hsa-miR-155-5p in predicting the outcome of patients with mCRC treated with B. In addition, the variation of circulating hsa-miR-155-5p could also be indicative of the patient survival.

## 1. Introduction

Targeting angiogenesis has been the standard of treatment in metastatic colorectal cancer (mCRC) for more than a decade, and novel anti-angiogenic agents are emerging each year. However, despite improvements in our understanding of the molecular biology of colorectal cancer (CRC), there are still no validated biomarkers for anti-angiogenic treatment. According to randomized clinical trials [1,2,3], bevacizumab (B), a monoclonal antibody against the vascular endothelial growth factor A, is widely used in combination with the chemotherapeutic regimen in a number of countries. Although several biomarkers have been studied and hypothesized to be useful for patient selection, none of these have yet been validated for use in clinical practice [4,5,6].

MicroRNAs (miRNAs) are a class of small non-coding RNAs approximately 18–25 nucleotides long with an important role in regulating gene expression. Expression patterns of miRNAs correlate with specific clinical pathological parameters in different cancer subtypes, suggesting that miRNAs could be potential biomarkers on the basis of tumor origin, histology, aggressiveness or chemosensitivity [7]. It has been reported that miRNAs may regulate the angiogenic process by exerting pro-angiogenic or anti-angiogenic effects [8,9,10,11,12,13,14]. Specific tumor tissue miRNAs have been shown to be predictive of the effectiveness of B in CRC patients [15]. The nature of miRNAs renders them particularly stable in biological fluids such as serum and plasma, making them potentially ideal circulating biomarkers for diagnosis, prognosis and as predictors of response to treatment [7,16]. With regard to this last characteristic, a miRNA signature composed of eight circulating miRNAs has been found to significantly correlate with overall survival (OS) in patients with glioblastoma treated with B [17], whereas a six-circulating miRNA signature has proven prognostic in patients with advanced non-small cell lung cancer treated with B plus erlotinib followed by platinum-based chemotherapy (CT) [18].

A previous study analyzed the role of circulating miR-126 in relation to outcome in patients treated with CT plus B. The authors demonstrated that an increased level of miR-126 from baseline to the first clinical evaluation was associated with a lack of benefit to treatment, concluding that it could represent a resistance mechanism to B [19].

In this study, we evaluated a panel of circulating miRNAs, including miR-126, selected on the basis of their role in the angiogenic process, as bio-markers of the treatment with bevacizumab. To this aim, we determined miRNA plasma levels in a case series of patients treated with a B-based CT, enrolled into the prospective multicentric randomized phase III study “Italian Trial in Advanced Colorectal Cancer” (ITACa) [20].

## 2. Results

### 2.1. Case Series

The clinical pathological characteristics of patients are shown in Table 1.

Median age was 65 years (range 37–83 years), and about two thirds (35 patients, 67.3%) were male, in a good performance status and in an advanced stage of the disease. The tumor localization was mainly the colon (71.1%), compared to the rectum (28.9%), and equally distributed as left- and right-sided tumors. A total of 48.1% had a *RAS* mutation (21 patients were *KRAS* mutated and four were *NRAS* mutated), and 11.5% had a *BRAF* mutation.

### 2.2. Baseline Circulating miRNAs in Relation to Clinical Pathological Characteristics of Patients

By GeNorm analysis, two miRNAs (hsa-miR-484 and hsa-miR-223-3p) resulted in being the more stable and were used for the normalization analysis together with the spike in cel-miR-39-3p.

Baseline circulating levels of some miRNAs were significantly associated with the clinical pathological characteristics of patients. Of the analyzed miRNAs, three (hsa-miR-199a-5p, hsa-miR-335-5p and hsa-miR-520d-3p) were significantly upregulated in left-sided compared to right-sided lesions (Table 2) and two were significantly correlated with *RAS* status.

In particular, hsa-miR-21-5p was significantly downregulated in both *KRAS* and *NRAS* mutated patients. Conversely, hsa-miR-221-3p was significantly upregulated in *RAS* mutated patients (Table 3).

### 2.3. Response to Therapy and Prognosis in Relation to Clinical Pathological Characteristics of Patients

Overall, an objective response rate (ORR) of 62.7% was observed. Progression-free (PFS) and overall survival (OS) were 9.7 months (95% confidence interval (CI) 8.1–14.1) and 22.7 months (95% CI 13.1–28.8), respectively. No correlation was found between response to therapy and clinical pathological characteristics of patients. Conversely, performance and *BRAF* statuses were significantly associated with both PFS and OS. In particular, a hazard ratio (HR) of 2.32 (95% CI 1.06–5.08), *p* = 0.036, and HR of 3.27 (95% CI 1.45–7.41), *p* = 0.004, were observed for performance status in relation to PFS and OS, respectively, whereas HR of 3.41 (95% CI 1.35–8.59), *p* = 0.009, and HR of 3.62 (95% CI 1.45–9.07), *p* = 0.006, were observed for *BRAF* status in relation to PFS and OS, respectively. Age, dichotomized on the basis of the median value, was significantly associated with PFS (HR 2.11 (95% CI 1.15–3.89), *p* = 0.016) but not with OS. Moreover, CT regimen was associated with OS: HR 1.99 (95% CI 1.05–3.79), *p* = 0.035 (Table S1).

### 2.4. Baseline Circulating miRNAs in Relation to Response to Therapy and Patient Prognosis

With regard to response to therapy, only hsa-miR-17-5p resulted in being significantly correlated, with an odds ratio (OR) of 0.87 (95% CI 0.77–0.99).

In univariate analysis, two miRNAs, hsa-miR-20b-5p and hsa-miR424-5p, were significantly associated with PFS and OS. In particular, HR of 0.931 (95% CI 0.880–0.986, *p* = 0.014) and of 0.932 (95% CI 0.869–0.999, *p* = 0.048) were observed for PFS. With regard to OS, HR of 0.922 (95% CI 0.869–0.978, *p* = 0.007) and of 0.891 (95% CI 0.827–0.960, *p* = 0.002) were observed. miRNA hsa-miR-29b-3p resulted in being significantly correlated with PFS (HR 0.868 (95% CI 0.796–0.948), *p* = 0.002), but not with OS (*p* = 0.070). In addition, hsa-miR-155-5p was borderline associated with PFS and OS (*p* = 0.078 and 0.065, respectively) (Table S2).

In multivariate analysis, considering miRNAs levels as continuous variables, hsa-miR-20b-5p, hsa-miR-29b-3p and hsa-miR-155-5p resulted in being significantly associated with PFS (*p* = 0.027, *p* = 0.034 and *p* = 0.039, respectively) and OS (*p* = 0.044, *p* = 0.024 and *p* = 0.032, respectively) (Table 4).

**Figure 1:**
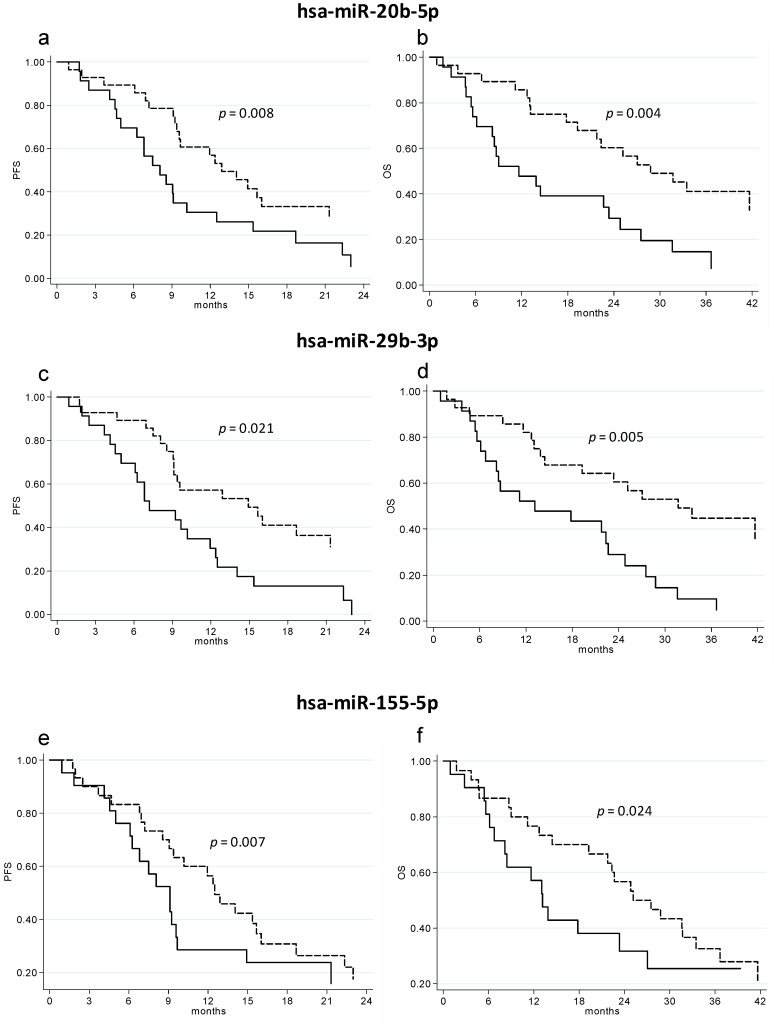
PFS and OS of basal circulating levels of hsa-miR-20b-5p (**a**,**b**), hsa-miR-29b-5p (**c**,**d**) and hsa-miR-155-5p (**e**,**f**). Dashed lines represent patients with miRNA values greater than the median value, whereas continuous lines represent patients with miRNA values lower than the median value.

Setting the median value as the cutoff, statistically-significant differences were seen in terms of PFS and OS for the three miRNAs. In particular, significantly longer PFS and OS were observed for patients with circulating miRNA values over the cutoff (Table 5 and Figure 1).

### 2.5. Circulating miRNAs’ Variations during Treatment in Relation to Patient Outcome

Variations of miRNAs at the first clinical evaluation with respect to baseline were analyzed. Differences in miRNAs variation were observed in relation to the clinical pathological characteristics of patients. In particular, different variations were observed in relation to left- or right-sided tumors for hsa-miR-16-5p (*p* = 0.049), hsa-miR-221-3p (*p* = 0.011), hsa-miR-29b-3p (*p* = 0.015) and hsa-miR-335-5p (*p* = 0.026). Variations of hsa-miR-221-3p were also associated with the *BRAF* status (*p* = 0.049). Moreover, hsa-miR-194-5p variations were significantly associated with the *KRAS* status (*p* = 0.040).

**Figure 2:**
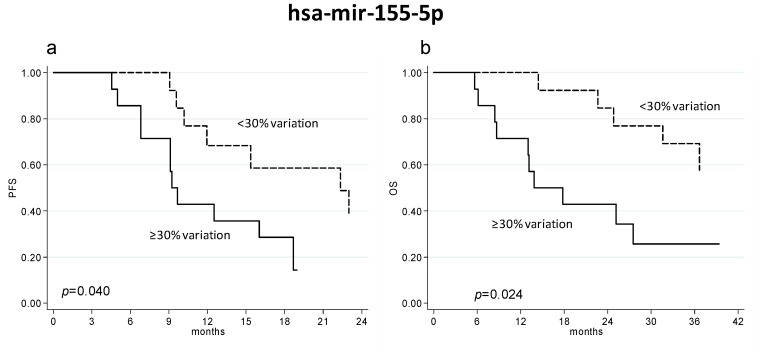
PFS (**a**) and OS (**b**) of patients with an increase ≥ or <30% of circulating hsa-miR-155-5p at the first clinical evaluation. Dashed lines represent patients with miRNA values greater than the median value, whereas continuous lines represent patients with miRNA values lower than the median value.

We analyzed the variation of circulating miRNA expression at the first clinical evaluation in relation to response to treatment and PFS and OS. No significant associations were observed between miRNA variations and response to therapy. Conversely, we observed that an increase of hsa-miR-155-5p was significantly associated with shorter PFS (HR 3.03 (95% CI 1.06–9.09), *p* = 0.040) and OS (HR 3.45 (95% CI 1.18–10.00), *p* = 0.024), with PFS and OS of 9.5 (95% CI 6.8–18.7) and 15.9 (95% CI 8.4– not reached), respectively, in patients with an increase ≥30% of hsa-miR-155-5p and 22.3 (95% CI 10.2–25.5) and 42.9 (24.8–not reached) months, respectively, in patients without such increase (Figure 2). An increase of hsa-miR-24-3p was also associated with a significantly shorter PFS (HR 2.22 (95% CI 0.99–5.00), *p* = 0.053), and OS (HR 2.13 (95% CI 0.89–5.00), *p* = 0.087).

## 3. Discussion

In this study, we found that specific circulating miRNAs are associated with prognosis in mCRC patients treated with CT plus B. Baseline circulating levels of hsa-miR-20b-5p, hsa-miR-29b-3p and hsa-miR-155-5p were significantly correlated with PFS and OS. Patients with higher baseline levels of the three miRNAs showed longer PFS and OS, suggesting that they could be involved in pathways potentially correlated with the angiogenic pathway and, as a consequence, with B efficacy. It has been demonstrated that hsa-miR-29b is capable of repressing tumor angiogenesis, invasion and metastasis, by targeting metalloproteinase-2 (MMP2) [21]. Similarly, another study has demonstrated that miR-29b in non-small cell lung cancer models could suppress cells proliferation, migration and invasion by targeting the 3’-UTR of MMP2 and PTEN mRNA [22]. More recently, it has been reported that hsa-miR-29b suppresses tumor growth through simultaneously inhibiting angiogenesis and tumorigenesis by targeting Akt3 [23]. These findings are in agreement with our results, suggesting that patients with higher levels of hsa-miR-29b could have a greater benefit from B as both exert an anti-angiogenic effect. Although little evidence is available on the correlation between miR-20b and the angiogenic process, a recent study reported the role of hsa-miR-20b in regulating proliferation and senescence of endothelial cells, through the involvement of RBL1 [24]. We also observed that patients with a high basal level of hsa-miR-155-5p had a better outcome and that patients with a rise in the level of this type of miRNA at the first clinical evaluation (i.e., after one month of treatment) had a considerable shorter PFS and OS. Of the many studies on the role of hsa-miR-155-5p in the angiogenic process [25,26,27], some have reported a role in the process of hypoxia [25,28], showing that hsa-miR-155 contributes to controlling hypoxia-inducible factor 1-alpha (HIF-1α) expression and promotes angiogenesis under hypoxia condition. The association between high basal levels of hsa-miR-155-5p and better outcome of patients treated with B is consistent with the link between this miRNA and angiogenesis and inflammation processes [27,29], both targets of the drug. On the other hand, the induction of circulating hsa-miR-155 after treatment with B could indicate a process of drug resistance due to the stimulation of angiogenesis that could contrast with the B activity and that could be in line with the poor prognosis of such patients. We also observed a less evident association between the induction of circulating hsa-miR-24-3p and a worse outcome. It has been shown that the endothelial nitric oxide synthase (eNOS) gene is one of the targets of hsa-miR-24-3p and that hsa-miR-24-3p inhibition increases eNOS protein expression [30,31] with a consequent role in the angiogenic process. In contrast with previous findings [19], our study did not reveal any correlation between miR-126 circulating levels and patient outcome.

We also showed that baseline circulating miRNA levels differed with respect to patient clinical pathological characteristics, in particular tumor location and *RAS* status. As defined previously [32], left-sided tumors (originating in the splenic flexure, descending colon, sigmoid colon, rectum or one-third of the transverse colon) derive from the embryonic hindgut, whereas right-sided tumors (originating in the appendix, cecum, ascending colon, hepatic flexure or two-thirds of the transverse colon) derive from the embryonic midgut. The hsa-miR-199a-5p, hsa-miR-335-5p and hsa-miR-520d-3p miRNAs were significantly more upregulated in patients with left-sided than right-sided lesions, reflecting the different tumor biology. As all three miRNAs act as tumor suppressors [33,34,35,36], their overexpression in left-sided tumors could partially explain the better outcome of this group of patients. These differences in circulating miRNA expression with respect to tumor side agree with our recent report indicating different gene expression profiles, inflammatory indexes and responses to B in patients with left- and right-sided tumors [37]. Furthermore, miRNAs has-miR-21-5p and hsa-miR-221-3p were found to be significantly correlated with RAS status. Decreased hsa-miR-21-5p and increased hsa-miR-221-3p were observed in RAS mutated patients with respect to *RAS* wt patients. Although it has been demonstrated that these miRNAs are involved in the angiogenic process, we did not observe a correlation between hsa-miR-21-5p or hsa-miR-221-3p and response to bevacizumab.

This study has some limitations. First, the sample size was small, making it necessary to confirm results in a larger case series. Moreover, the lack of a control group treated with chemotherapy alone did not permit us to understand whether miRNAs were of prognostic value or predictive of response to B. Finally, we restricted our analysis to a specific panel of miRNAs on the basis of literature results, but cannot exclude that other miRNAs may play a role in response to B.

## 4. Materials and Methods

### 4.1. Case Series

This study included patients enrolled in the ITACa clinical trial [17], randomized to be treated with first-line CT (FOLFOX4 or FOLFIRI) only or CT plus B. Fifty-two patients in the CT + B arm, whose biological material was available, were analyzed for this study. All patients were characterized for RAS and BRAF status by MassARRAY (Sequenom, San Diego, CA, USA) using the Myriapod Colon status (Diatech Pharmacogenetics, Jesi, Italy) as the routine diagnostic procedure. Consenting patients underwent periodic blood sampling: peripheral blood samples were collected at baseline (before treatment began), at the first evaluation (after around 2 months) and when progressive disease (PD) was documented. All patients were assessed for response, PFS and OS according to RECIST (Response Evaluation Criteria In Solid Tumors) criteria Version 1.1. Tumor response was evaluated every 2 months by CT scan. Responders included patients with a complete response (CR) and a partial response (PR). Non-responders included patients with stable disease (SD) or PD. The study protocol was approved by the Local Ethics Committee (Comitato Etico Area Vasta e Istituto Scientifico Romagnolo per lo Studio e la Cura dei Tumori, no. 674) on 19 September 2007. All patients gave informed consent before blood sample collection.

### 4.2. miRNA Selection

A review of the literature was made for selecting the panel of miRNAs for analysis in plasma. The miRNAs were selected on the basis of their role in the angiogenic process, especially in CRC, and evidence of their possible determination in plasma or serum [38,39,40]. Twenty-three miRNAs were selected for analysis as follows: has-miR-107, hsa-miR-126-3hashsa-miR-145-5p, hsa-miR-194-5p, hsa-miR-199a-5p, hsa-miR-200b-3p, hsa-miR-20b-5p, hsa-miR-21-5p, hsa-miR-210-3p, hsa-miR-221-3p, hsa-miR-223-3p, hsa-miR-24-3p, hsa-miR-27a-3p, hsa-miR-29b-3p, hsa-miR-335-5p, hsa-miR-424-5p, hsa-miR-484, hsa-miR-497-5p, hsa-miR-520d-3p, hsa-miR-92a-3p, hsa-miR-17-5p, hsa-miR-155-5p, hsa-miR-16-5p. Moreover, cel-miR-39-3p was used as a spike-in for exogenous normalization.

### 4.3. Circulating miRNA Expression Analysis

Plasma was obtained from peripheral blood collected in EDTA-tubes, after centrifugation at 3000 rpm for 15 min. Plasma samples were stored at −80 °C until miRNA extraction. miRNAs were extracted from 400 µL of plasma using the miRVANA PARIS kit (Thermofisher, Monza, Italy). The 24 selected miRNAs were then spotted in array custom plates. For the selection of housekeeping (HSK) miRNAs, results were analyzed by GeNorm software (v. 3.2) to evaluate the stablest miRNAs. Assays were run on a 7500 Real-Time PCR System (Thermofisher). The reactions were initiated at 95 °C for 5 min followed by 40 cycles of 95 °C for 15 s and 60 °C for 1 min. All reactions, including the no template controls, were run in duplicate. Data were analyzed using Expression suite software v1.1 (Thermofisher) according to the ΔΔ*C*t method.

### 4.4. Statistical Analysis

The aim of this analysis was to examine the association between baseline circulating miRNA expression levels and PFS, OS and ORR in the ITACa case series and to evaluate their modification during CT + B therapy. The primary objective of the ITACa trial was PFS. Secondary efficacy endpoints were ORR and OS. PFS was calculated as the time from the date of randomization to the date of the first documented evidence of PD (per investigator assessment), last tumor assessment or death in the absence of disease progression. Patients submitted to curative metastasectomy were censored at the time of surgery. OS was calculated as the time from the date of randomization to the date of death from any cause or last follow-up. Descriptive statistics were used to describe patients. The relationship between baseline miRNA expression and clinical pathological factors was evaluated using a nonparametric ranking statistic test (median test). The median value of variation in the case series (30%) was set as the cutoff point. Time-to-event data (PFS, OS) were described using the Kaplan–Meier method and compared using the log rank test (significance level of 5%). Ninety-five percent confidence intervals (95% CI) were calculated using nonparametric methods. Estimated HR and their 95% CI were calculated by the Cox regression model. The multivariate Cox regression model was used to select the most useful prognostic markers of all the miRNAs used (considered as continuous variables) adjusting for clinical pathological characteristics of patients statistically significant at univariate analysis. Circulating basal levels of miRNAs were dichotomized into “high” or “low” according to median values [41]. We also conducted landmark analyses to reduce possible confounding by time on treatment by assessing the impact of miRNA level change from baseline to first tumor evaluation (about 2 months of onset of the treatment protocol) of survival outcomes. Patients who were still alive and not lost to follow-up at the landmark time were divided into two categories, i.e., patients who had progressed and patients who had not progressed by that time. PFS and OS after the landmark time were computed using the Kaplan–Meier curves. Logistic regression models were used to assess OR and their 95% CI in order to evaluate the association between miRNA baseline levels and ORR (CR + PR). All *p*-values were based on two-sided testing, and statistical analyses were performed using SAS statistical software Version 9.4 (SAS Institute, Cary, NC, USA).

## 5. Conclusions

This study showed that circulating higher levels of hsa-miR-20b-5p, hsa-miR-29b-3p and hsa-miR-155-5p at baseline are associated with a better prognosis in mCRC patients treated with B-based CT. Measuring these miRNAs before treatment could be helpful in selecting the patients who are more likely to benefit from the drug. The increase in circulating hsa-miR-155-5p after one month of treatment is associated with much shorter PFS and OS, suggesting that the determination of this miRNA during treatment could give important information for monitoring the drug response. These results should be confirmed and verified in an independent case series before being translated into the clinical setting.

## Figures and Tables

**Table 1:** Patient characteristics (*n* = 52).

Patient Characteristics	No. (%)
Median age, years (range)	65 (37–83)
Gender	
Male	35 (67.3)
Female	17 (32.7)
Performance Status (ECOG)	
0	44 (84.6)
1–2	8 (15.4)
Stage at Diagnosis	
I–III	12 (23.1)
IV	40 (76.9)
Tumor Localization	
Colon	37 (71.1)
Rectum	15 (28.9)
Left-sided	27 (55.1)
Right-sided	22 (44.9)
Grading	
1–2	25 (59.5)
3	17 (40.5)
Missing	10 (19.0)
CT Regimen	
FOLFOX4	27 (51.9)
FOLFIRI	25 (48.1)
Prior Cancer Therapy	
Surgery	40 (76.9)
Radiotherapy	4 (7.7)
Adjuvant CT	9 (17.3)
*RAS* Status	
Wild type	27 (51.9)
Mutated	25 (48.1)
*BRAF* Status	
Wild type	46 (88.5)
Mutated	6 (11.5)

ECOG, Eastern Cooperative Oncology Group; CT: chemotherapy; FOLFOX4, folinic acid, 5-fluorouracil and oxaliplatin; FOLFIRI, folinic acid, 5-fluorouracil and irinotecan.

**Table 2:** miRNAs significantly correlated with tumor localization.

miRNA	Left-Sided	Right-Sided	*p*
	Median Value (Range)		
hsa-miR-199a-5p	3188 (0.5–149,395)	1960.5 (0.47–48,761)	0.034
hsa-miR-335-5p	6574.5 (1493–1,332,286)	3214 (2.14–40,038)	0.006
hsa-miR-520d-3p	5087 (3.09–2,831,724)	1505 (0.49–48,452)	0.008

**Table 3:** miRNAs significantly correlated with *RAS* status.

miRNA	*KRAS*		*p*	*NRAS*		*p*
	Median Value (Range)			Median Value (Range)		
	Wild Type	Mutated		Wild Type	Mutated	
hsa-miR-21-5p	1424 (0.57–4627)	1.71 (0.53–3594)	0.019	1558 (0.57–4627)	1011 (0.53–3594)	0.008
hsa-miR-221-3p	1163 (0.03–5499)	1878 (0.58–34,375)	0.050	1122 (0.03–5499)	1866 (0.58–34,375)	0.010

**Table 4:** Multivariate analysis of PFS and OS.

Baseline	PFS		OS	
	HR (95% CI)	*p*	HR (95% CI)	*p*
has-miR-20b-5p	0.922 (0.847–0.989)	0.035	0.930 (0.850–0.995)	0.046
has-miR-29b-3p	0.854 (0.728–0.997)	0.045	0.872 (0.753–0.991)	0.039
has-miR-424-5p	0.968 (0.877–1.069)	0.517	0.936 (0.838–1.046)	0.242
has-miR-155-5p	0.927 (0.863–0.997)	0.040	0.917 (0.850–0.990)	0.026
ECOG PS (1–2 vs. 0)	1.206 (0.424–3.433)	0.725	1.838 (0.667–5.060)	0.239
BRAF (mutated vs. wild type)	3.574 (1.075–11.882)	0.038	3.628 (1.063–12.378)	0.040
Age, years (≥65 vs. <65)	2.207 (0.987–4.935)	0.054	1.478 (0.650–3.364)	0.351

**Table 5:** Univariate analysis of PFS and OS in relation to miRNA cutoff values.

**PFS**	**No. Patients**	**No. Events**	**Median PFS (Months) (95% CI)**	***p***	**HR (95% CI)**	***p***
hsa-miR-20b-5p						
<1293	26	24	8.1 (5.0–12.5)		1.00	
≥1293	26	20	14.0 (9.4–21.3)	0.008	0.44 (0.24–0.82)	0.010
hsa-miR-29b-3p						
<3138	25	23	8.2 (5.0–12.4)		1.00	
≥3138	27	21	14.9 (9.1–21.3)	0.021	0.50 (0.27–0.91)	0.024
hsa-miR-155-5p						
<0.73	32	30	8.3 (6.1–9.7)		1.00	
≥0.73	20	14	16.0 (10.2–23.0)	0.007	0.42 (0.22–0.81)	0.009
**OS**	**No. Patients**	**No. Events**	**Median OS (Months) (95% CI)**	***p***	**HR (95% CI)**	***p***
hsa-miR-20b-5p						
<1293	26	23	11.6 (8.2–23.4)		1.00	
≥1293	26	17	28.8 (19.3–42.9)	0.004	0.40 (0.21–0.77)	0.005
hsa-miR-29b-3p						
<3138	25	22	15.5 (6.8–24.8)		1.00	
≥3138	27	18	31.7 (13.9–47.1)	0.005	0.40 (0.21–0.78)	0.007
hsa-miR-155-5p						
<0.73	32	27	13.5 (8.2–23.4)		1.00	
≥0.73	20	13	31.6 (21.8–42.9)	0.024	0.47 (0.24–0.92)	0.028

## Supplementary Materials

Supplementary materials can be found at www.mdpi.com/1422-0067/19/1/307/s1.

## Abbreviations

B	bevacizumab
CT	chemotherapy
eNOS	nitric oxide synthase
mCRC	metastatic colorectal cancer
miRNA	microRNA
MMP2	metalloproteinase-2
ORR	objective response rate
OS	overall survival
PFS	progression-free survival
