# Supplementary material for: Circulating Plasma Levels of miR-20b, miR-29b and miR-155 as Predictors of Bevacizumab Efficacy in Patients with Metastatic Colorectal Cancer
Source: Int J Mol Sci. 2018 Jan 20;19(1):307. doi: 10.3390/ijms19010307 (PMC5796251; doi:10.3390/ijms19010307)
Supplement: Supplementary file 1 [file ijms-19-00307-s001.pdf]

Supplementary Materials:

Table S1. PFS and OS in relation to patient clinical pathological characteristics.

	N.pts	Median PFS (months) (95% CI)	*p*	HR (95% CI)	*p*	Median OS (months) (95% CI)	*p*	HR (95% CI)	*p*
**Overall**	52	9.7 (8.1-14.1)	-	-	-	22.7 (13.1-28.8)	-	-	-
**Age (years)**									
<65	26	14.0 (9.1-25.1)		1.00		24.8 (13.9-41.7)		1.00	
≥65	26	7.9 (4.7-12.4)	0.014	2.11 (1.15-3.89)	0.016	18.6 (6.1-31.6)	0.199	1.50 (0.80-2.81)	0.202
**Gender**									
Female	17	9.2 (4.7-12.5)		1.00		13.6 (5.4-49.5)		1.00	
Male	35	11.9 (8.6-16.0)	0.379	0.75 (0.39-1.42)	0.381	24.8 (13.9-31.7)	0.572	0.82 (0.41-1.64)	0.573
**Performance Status (ECOG)**									
0	44	12.4 (9.1-15.7)		1.00		25.2 (17.8-33.5)		1.00	
1-2	8	7.2 (0.9-9.7)	0.031	2.32 (1.06-5.08)	0.036	10.2 (0.9-14.4)	0.003	3.27 (1.45-7.41)	0.004
**Stage at diagnosis**									
I-III	12	12.4 (1.9-21.3)		1.00		22.5 (11.1-47.1)		1.00	
IV	40	9.4 (7.2-14.9)	0.649	1.18 (0.58-2.40)	0.650	23.4 (9.0-31.6)	0.512	1.28 (0.61-2.71)	0.513
**Tumor localization**									
Rectum	15	11.9 (4.1-21.3)		1.00		27.1 (5.4-nr)		1.00	
Colon	37	9.3 (6.9-14.0)	0.415	1.32 (0.67-2.58)	0.417	20.5 (11.1-28.8)	0.155	1.70 (0.81-3.59)	0.160
Left-sided	27	12.4 (7.2-16.0)		1.00		22.3 (12.7-31.7)		1.00	
Right-sided	22	9.6 (6.9-15.7)	0.778	0.91 (0.49-1.70)	0.779	25.2 (9.0-33.5)	0.749	1.11 (0.58-2.11)	0.749
**Grading**									
1-2	25	12.9 (9.1-18.7)		1.00		28.8 (19.3-36.7)		1.00	
3	17	8.1 (5.0-14.9)	0.378	1.36 (0.68-2.72)	0.381	11.9 (6.8-27.5)	0.219	1.57 (0.76-3.24)	0.222
**CT regimen**									
FOLFOX4	27	12.7 (7.2-21.3)		1.00		28.8 (14.4-49.5)		1.00	
FOLFIRI	25	9.4 (6.8-12.4)	0.144	1.56 (0.85-2.87)	0.148	13.9 (11.1-24.8)	0.032	1.99 (1.05-3.79)	0.035
***RAS* status**									
Wild type	27	8.1 (6.1-12.5)		1.00		14.4 (8.2-31.7)		1.00	
Mutated	25	14.0 (9.2-18.7)	0.208	0.68 (0.37-1.25)	0.211	22.7 (13.9-47.1)	0.324	0.73 (0.39-1.37)	0.326
***BRAF* status**									
Wild type	46	11.9 (9.1-15.7)		1.00		24.8 (14.4-33.5)		1.00	
Mutated	6	6.5 (4.7-12.5)	0.006	3.41 (1.35-8.59)	0.009	7.6 (4.7-27.5)	0.003	3.62 (1.45-9.07)	0.006

Table S2. Univariate analysis of PFS and OS

	**PFS**		**OS**	
**Baseline**	**HR (95% CI)**	***p***	**HR (95% CI)**	***p***
hsa-miR-107	0.999 (0.932-1.071)	0.985	0.986 (0.919-1.057)	0.682
hsa-miR-126-3p	0.912 (0.807-1.031)	0.142	0.910 (0.804-1.029)	0.131
hsa-miR-145-5p	1.008 (0.958-1.060)	0.770	0.999 (0.949-1.052)	0.972
hsa-miR-16-5p	0.980 (0.929-1.034)	0.459	0.959 (0.907-1.014)	0.139
hsa-miR-194-5p	1.011 (0.944-1.082)	0.758	0.985 (0.919-1.055)	0.660
hsa-miR-199a-5p	1.009 (0.951-1.070)	0.769	0.994 (0.935-1.056)	0.836
hsa-miR-200b-3p	1.061 (0.976-1.154)	0.166	1.035 (0.951-1.125)	0.429
hsa-miR-20b-5p	0.931 (0.880-0.986)	0.014	0.922 (0.869-0.978)	0.007
hsa-miR-21-5p	0.981 (0.924-1.041)	0.522	0.985 (0.925-1.049)	0.637
hsa-miR-210-3p	0.981 (0.923-1.044)	0.551	0.973 (0.912-1.038)	0.401
hsa-miR-221-3p	1.025 (0.970-1.084)	0.380	1.035 (0.975-1.098)	0.261
hsa-miR-24-3p	0.998 (0.947-1.053)	0.944	1.000 (0.946-1.057)	0.999
hsa-miR-27a-3p	1.039 (0.976-1.105)	0.232	1.014 (0.954-1.077)	0.659
hsa-miR-29b-3p	0.868 (0.796-0.948)	0.002	0.929 (0.857-1.006)	0.070
hsa-miR-335-5p	0.980 (0.861-1.117)	0.766	0.942 (0.840-1.058)	0.312
hsa-miR-424-5p	0.932 (0.869-0.999)	0.048	0.891 (0.827-0.960)	0.002
hsa-miR-497-5p	0.958 (0.887-1.033)	0.265	1.006 (0.910-1.112)	0.910
hsa-miR-520d-3p	1.014 (0.957-1.075)	0.635	0.997 (0.937-1.061)	0.924
hsa-miR-92a-3p	1.003 (0.943-1.067)	0.919	0.972 (0.913-1.035)	0.376
hsa-miR17-5p	0.951 (0.897-1.008)	0.091	0.956 (0.900-1.015)	0.137
hsa-mir-155-5p	0.944 (0.885-1.007)	0.078	0.941 (0.882-1.004)	0.065
